# Obesity Modifies the Performance of Fibrosis Biomarkers in Nonalcoholic Fatty Liver Disease

**DOI:** 10.1210/clinem/dgab933

**Published:** 2021-12-31

**Authors:** Sami Qadri, Noora Ahlholm, Ida Lønsmann, Paola Pellegrini, Anni Poikola, Panu K Luukkonen, Kimmo Porthan, Anne Juuti, Henna Sammalkorpi, Anne K Penttilä, Roberta D’Ambrosio, Giorgio Soardo, Diana J Leeming, Morten Karsdal, Johanna Arola, Stergios Kechagias, Serena Pelusi, Mattias Ekstedt, Luca Valenti, Hannes Hagström, Hannele Yki-Järvinen

**Affiliations:** 1 Department of Medicine, University of Helsinki and Helsinki University Hospital, Helsinki, Finland; 2 Minerva Foundation Institute for Medical Research, Helsinki, Finland; 3 Nordic Bioscience, Biomarkers and Research, Herlev, Denmark; 4 VLVbio AB, Nacka, Sweden; 5 Yale School of Medicine, Yale University, New Haven, CT,USA; 6 Department of Gastrointestinal Surgery, Abdominal Center, University of Helsinki and Helsinki University Hospital, Helsinki, Finland; 7 Division of Gastroenterology and Hepatology, Fondazione IRCCS Ca’ Granda Ospedale Maggiore Policlinico, Milan, Italy; 8 Clinic of Internal Medicine—Liver Unit, Department of Medical Area (DAME), Università degli Studi di Udine, Udine, Italy; 9 Italian Liver Foundation, Area Science Park, Basovizza Campus, Trieste, Italy; 10 Department of Pathology, University of Helsinki and Helsinki University Hospital, Helsinki, Finland; 11 Department of Health, Medicine, and Caring Sciences, Linköping University, Linköping, Sweden; 12 Precision Medicine—Department of Transfusion Medicine and Hematology, Fondazione IRCCS Ca’ Granda Ospedale Maggiore Policlinico, Milan, Italy; 13 Department of Pathophysiology and Transplantation, Università degli Studi di Milano, Milan, Italy; 14 Department of Medicine, Huddinge, Karolinska Institutet, Stockholm, Sweden

**Keywords:** nonalcoholic fatty liver disease, nonalcoholic steatohepatitis, fibrosis, cirrhosis, biomarkers, obesity

## Abstract

**Context:**

Guidelines recommend blood-based fibrosis biomarkers to identify advanced nonalcoholic fatty liver disease (NAFLD), which is particularly prevalent in patients with obesity.

**Objective:**

To study whether the degree of obesity affects the performance of liver fibrosis biomarkers in NAFLD.

**Design:**

Cross-sectional cohort study comparing simple fibrosis scores [Fibrosis-4 Index (FIB-4); NAFLD Fibrosis Score (NFS); aspartate aminotransferase to platelet ratio index; BARD (body mass index, aspartate-to-alanine aminotransferase ratio, diabetes); Hepamet Fibrosis Score (HFS)] and newer scores incorporating neo-epitope biomarkers PRO-C3 (ADAPT, FIBC3) or cytokeratin 18 (MACK-3).

**Setting:**

Tertiary referral center.

**Patients:**

We recruited overweight/obese patients from endocrinology (n = 307) and hepatology (n = 71) clinics undergoing a liver biopsy [median body mass index (BMI) 40.3 (interquartile range 36.0-44.7) kg/m^2^]. Additionally, we studied 859 less obese patients with biopsy-proven NAFLD to derive BMI-adjusted cutoffs for NFS.

**Main Outcome Measures:**

Biomarker area under the receiver operating characteristic (AUROC), sensitivity, specificity, and predictive values to identify histological stage ≥F3 fibrosis or nonalcoholic steatohepatitis with ≥F2 fibrosis [fibrotic nonalcoholic steatohepatitis (NASH)].

**Results:**

The scores with an AUROC ≥0.85 to identify ≥F3 fibrosis were ADAPT, FIB-4, FIBC3, and HFS. For fibrotic NASH, the best predictors were MACK-3 and ADAPT. The specificities of NFS, BARD, and FIBC3 deteriorated as a function of BMI. We derived and validated new cutoffs for NFS to rule in/out ≥F3 fibrosis in groups with BMIs <30.0, 30.0 to 39.9, and ≥40.0 kg/m^2^. This optimized its performance at all levels of BMI. Sequentially combining FIB-4 with ADAPT or FIBC3 increased specificity to diagnose ≥F3 fibrosis.

**Conclusions:**

In obese patients, the best-performing fibrosis biomarkers are ADAPT and the inexpensive FIB-4, which are unaffected by BMI. The widely used NFS loses specificity in obese individuals, which may be corrected with BMI-adjusted cutoffs.

Most patients with nonalcoholic fatty liver disease (NAFLD) are either overweight or obese (1). Obesity is a key predictor of advanced liver fibrosis (bridging fibrosis [stage F3] or cirrhosis [stage F4]), which is associated with over a 3-fold increase in the risk of all-cause and liver-related mortality (2,3). Thus, timely identification of affected patients by obesity-treating clinicians in both primary care and specialist clinics is essential, as even advanced fibrosis is amenable to weight loss (4). Furthermore, the substantial long-term resolution of liver fibrosis in patients undergoing bariatric surgery underlines the importance of case finding for prioritizing those who will benefit most from invasive treatment strategies (5).

Guidelines recommend first-line screening of advanced fibrosis using composite biomarker scores, primarily the Fibrosis-4 Index (FIB-4) and the NAFLD Fibrosis Score (NFS) (6-9). Other less commonly used scores include the aspartate aminotransferase to platelet ratio index (APRI) (10), BARD [body mass index (BMI), aspartate-to-alanine aminotransferase ratio, diabetes] (11), and the recently developed Hepamet Fibrosis Score (HFS) (12), These biomarkers are inexpensive as they utilize readily available clinical information and routine laboratory tests. Recently, novel composite scores incorporating direct biomarkers of active fibrogenesis, such as the N-terminal type III collagen propeptide (PRO-C3; included in the ADAPT and FIBC3 scores), show utility in detecting advanced fibrosis associated with NAFLD (13,14). On the other hand, cytokeratin 18 (CK-18) antibodies M65 and M30 may be better biomarkers of nonalcoholic steatohepatitis (NASH) than of advanced fibrosis (15). MACK-3 (homeostasis model assessment, aspartate aminotransferase, CK-18) is a score incorporating CK-18 and is specifically designed to predict the presence of fibrotic NASH, defined as active NASH with concomitant stage F2 or F3 fibrosis (16).

Data are sparse regarding the applicability of fibrosis biomarkers in patients with morbid obesity. To our knowledge, previous studies have included 88 to 331 patients and mostly examined either NFS or BARD, both of which showed variable specificity (17-22). A limitation of most fibrosis biomarker studies is the inclusion of patients solely from hepatology clinics, with lower rates of severe obesity in comparison with obesity and diabetes clinics. As the highest risk of NAFLD and liver fibrosis is among the obese and metabolically unhealthy, however, noninvasive biomarkers should be specifically examined in these populations (7,23,24). Furthermore, as the NFS, BARD, and FIBC3 scores rely heavily on BMI as a predictor variable, it is unclear whether an increase in false positives could occur when they are applied in populations mainly comprising obese individuals. No single study has compared a wide array of old and new biomarkers in a large obese cohort with histological data. More important, the effect of BMI on the performance of fibrosis biomarkers remains unknown.

A limitation of simple scores such as FIB-4 and NFS is their relatively low diagnostic accuracy, mandating the use of 2 cutoff points to rule in or rule out advanced fibrosis and leaving up to 50% of all patients in an indeterminate gray area (9,25,26). In such cases, fibrosis should be evaluated by more accurate tests such as transient elastography, which is, however, seldom available outside of tertiary centers due to cost and operator training requirements (27). A more feasible alternative could be another blood-based test. A large prospective study recently showed that use of the proprietary enhanced liver fibrosis test in patients with elevated liver enzymes and an indeterminate FIB-4 reduced unnecessary referrals to diagnose advanced fibrosis in NAFLD (28). There are no data on whether sequential use of existing or novel blood-based biomarkers could be of benefit in screening for advanced fibrosis in patients with obesity.

Our aims were to find the best-performing biomarkers of advanced fibrosis and fibrotic NASH in obese individuals and to study whether biomarker performance is affected by BMI. Additionally, we studied whether a sequential mode of testing increases diagnostic yield for advanced fibrosis. To this end, we recruited 378 overweight/obese patients with a liver biopsy. We compared simple composite scores (FIB-4, NFS, APRI, BARD, HFS) to direct neo-epitope biomarkers of fibrogenesis (PRO-C3) and hepatocellular injury (CK-18 M65/M30), as well as scores derived from PRO-C3 (ADAPT, FIBC3) and CK-18 (MACK-3). Because BMI significantly modified the performance of NFS, we studied an additional 859 patients from external cohorts to validate new BMI-adjusted cutoffs.

## Materials and Methods

### Patients and Study Design

#### Overweight/obese cohort

The cohort comprised 378 patients studied at the Helsinki University Hospital (Helsinki, Finland). Of these, 307 had a BMI of >30 kg/m^2^ and were consecutively recruited among those eligible for weight-loss surgery. The other 71 had a BMI of >25 kg/m^2^ and were referred to the gastroenterologist due to elevated liver enzymes and suspected NASH. All patients fulfilled the following inclusion criteria: (1) age 18 to 75 years; (2) no evidence of acute or chronic disease except for obesity, hypertension, dyslipidemia, NAFLD, or type 2 diabetes (T2DM) based on history, physical examination, standard laboratory tests (complete blood counts, serum creatinine, thyrotropin, and electrolyte concentrations), and electrocardiogram; (3) alcohol consumption < 20 g per day for women and < 30 g per day for men; (4) no use of drugs or toxins associated with steatosis; and (5) not pregnant or lactating. A week before undergoing a liver biopsy, the patients arrived at the Clinical Research Unit after an overnight fast. A history and physical examination were performed, including measurement of body weight and height (29). Fasting blood samples were drawn for standard biochemical measurements, as well as for measurement of concentrations of PRO-C3 and CK-18 M65/M30 (29). A wedge biopsy of the liver was taken from the 307 patients undergoing bariatric surgery, while percutaneous needle biopsies were taken from the 71 patients seen by a gastroenterologist. The Ethical Review Committee of the Hospital District of Helsinki and Uusimaa (Helsinki, Finland) approved the study.

#### External cohorts

Since we found NFS to be affected by BMI (see Results section), we recruited 2 additional cohorts from Swedish and Italian hepatology clinics for derivation and validation of new BMI-adjusted cutoffs.

The Swedish cohort comprised 646 patients from a cohort study with retrospectively collected data, including patients diagnosed with biopsy-proven NAFLD at the Karolinska University Hospital (Huddinge, Sweden) and Linköping University Hospital (Linköping, Sweden) from 1971 to 2009. Patients with liver disease other than NAFLD or diagnosis with any concurrent liver disease during follow-up were excluded. Otherwise, inclusion and exclusion criteria follow those of the overweight/obese cohort and have been previously published (30). The regional ethics committees at Karolinska Institutet and Linköping University approved the study.

The Italian cohort comprised 213 individuals who underwent a liver biopsy between 2016 and 2018 for suspected NASH, due to the presence of metabolic cofactors and evidence of steatosis at imaging plus persistently increased liver enzymes, ferritin, noninvasive predictors of liver fibrosis, or increased liver stiffness values (≥7.9 kPa). Part of this cohort has previously been described (31). Other causes of liver disease were ruled out, including high alcohol intake (≥30/20 g per day for males/females), viral and autoimmune hepatitis, hereditary hemochromatosis, and alpha-1-antitrypsin deficiency. Patients with hepatocellular carcinoma and current use of steatosis-inducing drugs were excluded. The regional ethics committee at the Fondazione IRCCS Ca’ Granda approved the study.

### Liver Histology (Reference Standard and Target Conditions)

Slides were coded and read at each clinical center by a single expert pathologist (J.A. in the overweight/obese cohort), blinded to the patients’ identities and histories. Histopathological features of NAFLD (steatosis, ballooning, lobular inflammation, fibrosis) were assessed using the NASH Clinical Research Network grading and definitions (32). Fibrosis stage was scored on a 5-point scale (F0-F4) (32). We diagnosed NASH when steatosis, lobular inflammation, and ballooning were concomitantly present (33). Fibrotic NASH was defined as NASH with a NAFLD Activity Score ≥ 4 and concomitant ≥ F2 fibrosis (34).

### Fibrosis Biomarkers (Index Tests)

Circulating neo-epitope biomarkers of fibrogenesis (PRO-C3) and cell death (CK-18 M65/M30) were measured in the overweight/obese cohort by personnel blinded to clinical data. PRO-C3 (a marker of interstitial matrix collagen type III formation) was measured in serum using competitive enzyme-linked immunosorbent assays (ELISAs; Nordic Bioscience A/S, Denmark) (35). Concentrations of caspase-cleaved CK-18 fragments were determined by measuring the M30 antibody (M30 Apoptosense CK18 kit [ELISA]; VLVbio, Nacka, Sweden), and the combination of cleaved and intact CK-18 using the M65 antibody (M65 ELISA CK-18 kit; VLVbio). We used clinical and laboratory data obtained at the clinical visit to calculate the FIB-4 (6), NFS (7), APRI (10), BARD (11), HFS (12), ADAPT (13), FIBC3 (14), and MACK-3 (16) scores, using formulae shown in Supplementary Table 1 (36). Cutoff values for advanced fibrosis follow those previously published, as outlined in Supplementary Table 2 (36). We used the age-adjusted cutoffs for FIB-4 and NFS in patients aged ≥65 years or older, where indicated in tables and figures (37).

### Statistical Analyses

We determined the overall diagnostic performance of fibrosis biomarkers and composite scores using the receiver operating characteristic (ROC) curve analysis as well as the area under the ROC curve (AUROC). The method by DeLong et al was used to compare AUROCs (38). To overcome ordinal scale and spectrum effect issues associated with the use of AUROC in the context of fibrosis stage, as suggested by Lambert et al (39), we calculated the Obuchowski measure (40). The measure can be interpreted as the probability that the biomarker will correctly rank 2 randomly chosen patients who have different fibrosis stages. A weighted penalty function of 0.25 per a difference of 1 unit in fibrosis stage was used. Sensitivity, specificity, positive predictive value (PPV), and negative predictive value (NPV), as well as positive likelihood ratio (LR+) and negative likelihood ratio (LR−) for each composite score and diagnostic algorithm were derived, along with their confidence intervals (CIs). When reporting the combined performance metrics of a test with 2 cutoffs, we considered indeterminate results as true positives/negatives (the patients were assumed to be ultimately diagnosed by biopsy with 100% accuracy). The number needed to diagnose was calculated as the inverse of the Youden’s index (41). Sensitivities and specificities of the composite scores were compared using the exact McNemar’s test for paired binomial responses or the Chi-squared test for unpaired data. PPVs and NPVs were compared using weighted generalized score test statistics. A *P*-value < 0.05 was considered statistically significant. In the few cases where data were insufficient to derive the full composite score, we report the number of patients in table and figure legends. Only patients with full data to derive a given score were included in each analysis.

To derive new BMI-adjusted cutoffs for NFS (see Results section), we divided the patients into groups who were either nonobese (BMI < 30.0 kg/m^2^), obese (BMI 30.0-39.9 kg/m^2^), or morbidly obese (BMI ≥ 40.0 kg/m^2^). Patients in each group were randomly split in a 70:30 ratio into derivation and validation groups. The single-best cutoff to diagnose advanced fibrosis was defined as the point having the highest Youden’s index in the derivation groups. We additionally determined a priori that to either rule out or rule in advanced fibrosis in an at-risk population with a prevalence of 15% (as shown by recent epidemiological data for patients with T2DM (42)), 2 cutoffs should be fixed at approximately 85% sensitivity and 95% specificity to achieve an NPV ≥ 95% or a PPV ≥ 75% (and an accuracy ≥ 90%), respectively. Similar sensitivity and specificity were shown for the original NFS cutoffs by Angulo et al (7). We performed replication by applying the new cutoffs in the validation groups.

Analyses were performed using R 4.0.3 or GraphPad Prism 9.1.0 (GraphPad Software, La Jolla, CA, USA).

## Results

### Characteristics of the Patients

Table 1 shows characteristics of the individual cohorts as well as of all patients (n = 1237). In the overweight/obese cohort, median BMI was 40.3 (interquartile range 36.0-44.7) kg/m^2^, ranging from 25.9 to 75.1 kg/m^2^. In the Swedish and Italian cohorts, median BMIs were 28.0 (25.7-30.8) kg/m^2^ and 29.8 (27.3-33.1) kg/m^2^ (combined BMI range 18.1-46.7 kg/m^2^). Despite having a significantly lower prevalence of histologically verified advanced fibrosis (F3-F4), the overweight/obese cohort had significantly higher NFS and BARD scores compared to the Swedish cohort and similar scores compared to the Italian cohort. In patients with advanced fibrosis compared to those with none-to-moderate fibrosis (F0-F2), all fibrosis biomarkers were significantly elevated [Supplementary Tables 3-6 (36)].

**Table 1.** Clinical characteristics of the patients

	Overweight/obese cohort	Swedish cohort	Italian cohort	All patients
n	378	646	213	1237
Age, years	50 ± 9	48 ± 14^a^	61 ± 10^a^^,^^b^	51 ± 13
Males, n (%)	110 (29)	402 (62)^a^	129 (61)^a^	641 (52)
BMI, kg/m^2^	40.3 (36.0, 44.7)	28.0 (25.7, 30.8)^a^	29.8 (27.3, 33.1)^a^^,^^b^	30.2 (26.8, 36.7)
Waist circumference, cm	120 (110, 131)	NA	NA	NA
Waist-to-hip ratio	0.95 (0.89, 1.01)	NA	NA	NA
fP-Glucose, mmol/L	6.1 ± 1.3	6.0 ± 2.2^a^	6.6 ± 1.9^a^^,^^b^	6.1 ± 1.9
B-HbA1c, %	6.0 ± 0.9	NA	NA	NA
fS-Insulin, mU/L	15 ± 11	NA	28 ± 50^a^	NA
HOMA-IR	3.15 (1.84, 4.93)	NA	5.33 (3.01, 7.66)^a^	NA
fP-Total cholesterol, mmol/L	4.2 ± 1.1	6.0 ± 1.4	4.9 ± 2.8^a^	4.5 ± 1.9
fP-HDL cholesterol, mmol/L	1.22 ± 0.36	NA	1.32 ± 0.42^a^	NA
fP-LDL cholesterol, mmol/L	2.6 ± 0.9	NA	2.8 ± 2.7^a^	NA
fP-Triglycerides, mmol/L	1.45 ± 1.13	2.35 ± 1.65	1.62 ± 0.81^a^	1.51 ± 1.03
P-ALT, U/L	43 ± 35	84 ± 52^a^	46 ± 28^a^^,^^b^	65 ± 48
P-AST, U/L	35 ± 21	50 ± 34^a^	38 ± 19^a^^,^^b^	44 ± 29
P-AST/ALT	1.0 ± 0.7	0.7 ± 0.7^a^	0.9 ± 0.3^b^	0.8 ± 0.7
P-GGT, U/L	59 ± 107	109 ± 127	84 ± 97^a^^,^^b^	68 ± 104
P-ALP, U/L	73 ± 36	91 ± 47	NA	NA
P-Albumin, g/L	38 ± 4	42 ± 4^a^	44 ± 5^a^^,^^b^	41 ± 5
B-Platelets, E10^9^	251 ± 63	247 ± 73	181 ± 86^a^^,^^b^	237 ± 77
Type 2 diabetes, n (%)	161 (43)	93 (14)^a^	119 (56)^a^^,^^b^	373 (30)
Fibrosis stage (F0/F1/F2/F3/F4), n	204/125/21/17/11	163/256/149/58/20^a^	25/30/39/40/79^a^^,^^b^	392/411/209/115/110
NASH, %	15	66^a^	53^a^^,^^b^	24
Direct biomarkers				
PRO-C3, ng/mL	11.0 (8.8, 14.2)	NA	NA	NA
CK-18 M30, U/L	169 (122, 257)^c^	NA	NA	NA
CK-18 M65, U/L	216 (154, 376)^d^	NA	NA	NA
Composite scores				
FIB-4	1.05 (0.78, 1.40)	0.94 (0.66, 1.43)^a^	1.93 (1.34, 3.08)^a^^,^^b^	1.09 (0.77, 1.66)
NFS	−0.23 (−1.26, 0.69)^e^	−2.26 (−3.22, −1.30)^a^	0.06 (−0.98, 1.03)^b^	−1.29 (−2.58, 0.08)
APRI	0.32 (0.24, 0.45)	0.40 (0.29, 0.64)^a^	0.48 (0.34, 0.80)^a^^,^^b^	0.38 (0.28, 0.59)
BARD	3.0 (2.0, 4.0)	1.0 (0.0, 2.0)^a^	3.0 (2.0, 4.0)^b^	2.0 (1.0, 3.0)
HFS	0.09 (0.03, 0.21)^f^	NA	NA	NA
ADAPT	5.14 (4.29, 5.87)	NA	NA	NA
FIBC3	−1.03 (−2.05, 0.18)	NA	NA	NA
MACK-3	0.10 (0.04, 0.24)^g^	NA	NA	NA

Data are in means ± SD, medians (25th, 75th percentiles), counts (percentages), or percentages. The Mann-Whitney U and Chi-squared tests were used for statistical testing.

Abbreviations: ALP, alkaline phosphatase; ALT, alanine aminotransferase; APRI, aspartate aminotransferase to platelet ratio index; AST, aspartate aminotransferase; B, blood; BMI, body mass index; CK-18, cytokeratin 18; f, fasting; GGT, gamma-glutamyltransferase; FIB-4, Fibrosis-4 Index; HbA1c, glycated hemoglobin A1c; HDL, high-density lipoprotein; HFS, Hepamet Fibrosis Score; HOMA-IR, homeostatic model assessment of insulin resistance; LDL, low-density lipoprotein; NA, not available; NASH, nonalcoholic steatohepatitis; NFS, NAFLD Fibrosis Score; P, plasma; PRO-C3, N-terminal type III collagen propeptide; S, serum.

^a^ *P* < 0.05 vs overweight/obese cohort.

^b^ *P* < 0.05 vs Swedish cohort.

^c^n = 354.

^d^n = 361.

^e^n = 373.

^f^n = 371.

^g^n = 352.

### Performance of Fibrosis Biomarkers in the Overweight/Obese Cohort

The ROC curves for all biomarkers are shown in Supplementary Figure 1 (36).

#### Advanced (≥F3) and significant (≥F2) fibrosis

The biomarkers that reached an AUROC of ≥0.85 for advanced fibrosis were ADAPT, FIB-4, FIBC3, and HFS (Table 2). ADAPT and FIB-4 had significantly higher AUROCs both (0.89) compared to most others. They also had the highest AUROCs of 0.82 and 0.79 for significant fibrosis (Table 2). The Obuchowski measure, which compares the discriminatory ability of the biomarkers between any 2 fibrosis stages, was highest for ADAPT (0.904) (Table 2).

**Table 2.** Overall diagnostic accuracy of the biomarkers in the overweight/obese cohort (n = 378)

Biomarker	Advanced fibrosis (≥F3)	Significant fibrosis (≥F2)	Fibrotic NASH	Obuchowski measure (95% CI)
	AUROC (95% CI)	AUROC (95% CI)	AUROC (95% CI)	
ADAPT	0.89 (0.82-0.95)^b^^,^^c^^,^^d^^,^^e^^,^^f^^,^^g^^,^^h^	0.82 (0.76-0.88)^b^^,^^c^^,^^d^^,^^e^	0.89 (0.84-0.94)^a^^,^^c^^,^^d^	0.904 (0.888-0.920)
FIB-4	0.89 (0.83-0.95)^b^^,^^c^^,^^d^^,^^e^^,^^f^^,^^g^^,^^h^	0.79 (0.72-0.85)^d^	0.82 (0.73-0.92)^d^	0.890 (0.875-0.905)
FIBC3	0.86 (0.79-0.93)^d^^,^^e^^,^^f^^,^^g^^,^^h^	0.78 (0.72-0.84)^d^	0.80 (0.72-0.88)^d^	0.890 (0.872-0.908)
HFS^j^	0.85 (0.77-0.93)^d^^,^^g^^,^^h^	0.77 (0.71-0.84)^d^	0.83 (0.74-0.93)^d^	0.891 (0.873-0.910)
APRI	0.82 (0.73-0.91)^h^	0.74 (0.66-0.82)	0.83 (0.72-0.94)^d^	0.882 (0.862-0.901)
PRO-C3	0.78 (0.67-0.89)	0.73 (0.64-0.82)	0.82 (0.73-0.92)^d^	0.885 (0.864-0.905)
NFS^k^	0.77 (0.68-0.86)^h^	0.68 (0.60-0.76)	0.65 (0.53-0.77)	0.862 (0.842-0.883)
CK-18 M65^l^	0.76 (0.66-0.86)	0.74 (0.65-0.82)	0.91 (0.87-0.95)^a^^,^^c^^,^^d^	0.884 (0.863-0.906)
CK-18 M30^m^	0.75 (0.64-0.86)	0.75 (0.67-0.83)	0.89 (0.83-0.95)^d^	0.884 (0.862-0.906)
MACK-3^i^^,^^n^	0.71 (0.57-0.84)	0.73 (0.64-0.83)	0.92 (0.86-0.97)^a^^,^^c^^,^^d^	0.886 (0.859-0.913)
BARD	0.63 (0.52-0.73)	NS	NS	0.828 (0.806-0.850)

The DeLong’s test for 2 correlated receiver operating characteristic curves was used for statistical testing.

Abbreviations: APRI, aspartate aminotransferase to platelet ratio index; AUROC, area under the receiver operating characteristic; CK-18, cytokeratin 18; FIB-4, Fibrosis-4 Index; HFS, Hepamet Fibrosis Score; NASH, nonalcoholic steatohepatitis; NFS, NAFLD Fibrosis Score; NS, not significant; PRO-C3, N-terminal type III collagen propeptide.

^a^ *P* < 0.05 vs FIBC3.

^b^ *P* < 0.05 vs APRI.

^c^ *P* < 0.05 vs PRO-C3.

^d^ *P* < 0.05 vs NFS.

^e^ *P* < 0.05 vs CK-18 M65.

^f^ *P* < 0.05 vs CK-18 M30.

^g^ *P* < 0.05 vs MACK-3.

^h^ *P* < 0.05 vs BARD.

^i^MACK-3 was developed specifically for fibrotic NASH.

^j^n = 371.

^k^n = 373.

^l^n = 361.

^m^n = 354.

^n^n = 352.

Table 3 shows performance of the composite scores by applying the previously published cutoffs for advanced fibrosis. ADAPT, which uses a single cutoff and therefore classified no patients as indeterminate, had the highest combined sensitivity (79%) and specificity (87%) as well as the lowest number needed to diagnose (1.5). Of the scores requiring use of 2 cutoffs, FIB-4 (sensitivity 82%, specificity 98%) and HFS (sensitivity 86%, specificity 97%) performed equally, classifying 28% and 30% of the patients as indeterminate, while NFS had a lower specificity (78%) and classified 54% of the patients as indeterminate (Table 3).

**Table 3.** Diagnostic performance of the composite scores to identify advanced fibrosis or fibrotic NASH in the overweight/obese cohort (n = 378)

Biomarker	Cutoff	Se, % (95% CI)	Sp, % (95% CI)	PPV, % (95% CI)	NPV, % (95% CI)	LR+	LR−	NND	Indeterminate,^e^ %
Advanced fibrosis									
ADAPT	6.3287	79 (59-92)	87 (83-91)	33 (22-46)	98 (96-99)	6.08	0.24	1.5	NA
FIBC3	0.4	61 (41-78)	84 (80-88)	24 (14-35)	96 (94-98)	3.81	0.46	2.2	NA
FIB-4	1.3 (2.0)^a^	82 (63-94)	72 (67-76)	19 (12-27)	98 (96-99)	2.93	0.25	1.9	28
	2.67	36 (19-56)	98 (96-99)	62 (35-85)	95 (92-97)	18.00	0.65	2.9	
NFS^b^	−1.455 (0.12)^a^	89 (72-98)	22 (18-27)	9 (6-12)	96 (89-99)	1.14	0.50	9.1	54
	0.676	61 (41-78)	78 (73-82)	18 (11-27)	96 (93-98)	2.77	0.50	2.6	
HFS^c^	0.12	86 (67-96)	67 (62-72)	18 (12-25)	98 (96-100)	2.61	0.21	1.9	30
	0.47	50 (31-69)	97 (94-98)	54 (33-73)	96 (93-98)	16.67	0.52	2.1	
APRI	1	32 (16-52)	98 (96-99)	60 (32-84)	95 (92-97)	16.00	0.69	3.3	NA
BARD	2	93 (76-99)	26 (21-31)	9 (6-13)	98 (92-100)	1.26	0.27	5.3	NA
Fibrotic NASH									
MACK-3^d^	0.134	100 (79-100)	64 (58-69)	13 (8-20)	100 (98-100)	2.78	0.00	1.6	32
	0.549	56 (30-80)	95 (92-97)	36 (19-58)	98 (95-99)	11.20	0.46	2.0	

Abbreviations: APRI, aspartate aminotransferase to platelet ratio index; FIB-4, Fibrosis-4 Index; HFS, Hepamet Fibrosis Score; LR+, positive likelihood ratio; LR−, negative likelihood ratio; NA, not applicable; NASH, nonalcoholic steatohepatitis; NFS, NAFLD Fibrosis Score; NND, number needed to diagnose; NPV, negative predictive value; PPV, positive predictive value; Se, sensitivity; Sp, specificity.

^a^In parentheses are cutoffs that are used for patients aged ≥ 65 years.

^b^n = 373.

^c^n = 371.

^d^n = 352.

^e^Proportion of patients with an indeterminate result (between the upper and lower cutoffs).

#### Fibrotic NASH

**Figure 1.**
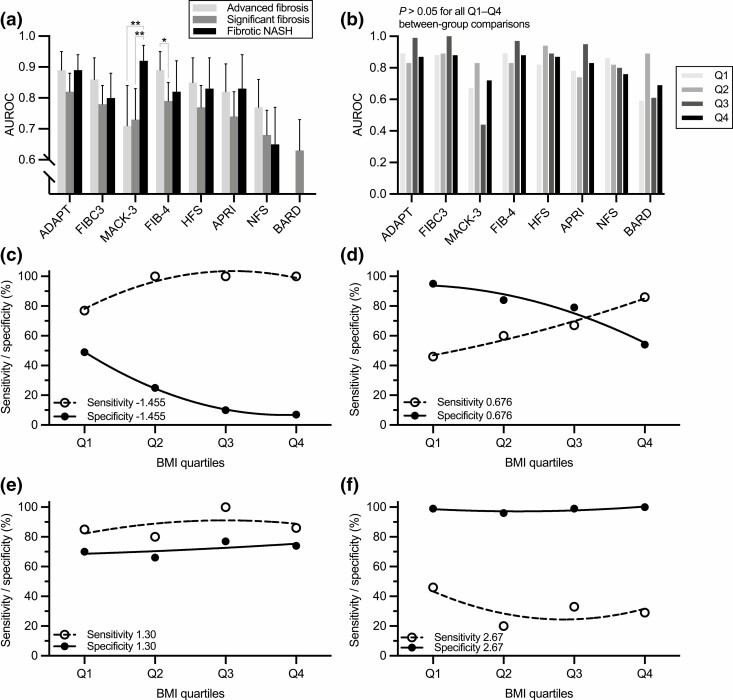
Performance of fibrosis biomarkers in the overweight/obese cohort (n = 378). (A) Areas under the receiver operating characteristic (AUROCs) for the composite scores to identify advanced fibrosis (F3-F4), significant fibrosis (F2-F4), or fibrotic nonalcoholic steatohepatitis (NASH + NAFLD Activity Score ≥ 4 + ≥F2). Whiskers denote 95% CI. The Delong’s test was used. *P < 0.05; **P < 0.01. (B) AUROCs for the composite scores to identify advanced fibrosis, based on groups divided by body mass index (BMI) quartiles (Q1-Q4). The DeLong’s test was used. (C-F) Sensitivities and specificities for the (C) lower and (D) upper cutoffs of the NAFLD Fibrosis Score (−1.455 and 0.676), and for the (E) lower and (F) upper cutoffs of the Fibrosis-4 Index (1.30 and 2.67) for advanced fibrosis, in groups divided based on BMI quartiles (Q1-Q4). Black circles and solid lines denote specificity, and white circles and dashed lines denote sensitivity. Regression lines were fitted using a quadratic model for visualization purposes.

The biomarkers that reached an AUROC of ≥0.85 for fibrotic NASH were MACK-3, CK-18 M65/M30, and ADAPT (Table 2). Both CK-18 M65 and M30, along with the MACK-3 score, were the only biomarkers that had a significantly higher AUROC for fibrotic NASH compared to advanced or significant fibrosis (Fig. 1A). Being the only biomarker with validated cutoffs (16), MACK-3 diagnosed or ruled out fibrotic NASH with a high degree of sensitivity (100%) and specificity (95%) (Table 3). Those classified as indeterminate comprised 32%.

#### Effect of BMI on biomarker performance

To examine whether BMI affects the diagnostic performance of fibrosis biomarkers, we next divided the overweight/obese cohort into groups based on BMI quartiles [median BMIs, Q1: 32.6 (27.5-34.4), Q2: 38.2 (37.3-39.2), Q3: 42.3 (41.1-43.4), Q4: 48.9 (46.4-51.6) kg/m^2^] [Supplementary Table 7 (36)]. The AUROCs of all biomarkers for advanced fibrosis were unchanged across the BMI quartiles (Fig. 1B, shown for the scores only). However, BMI substantially influenced the sensitivities and specificities of several composite scores. Most notably, the specificity of NFS linearly decreased as a function of BMI (Fig. 1C and 1D). A similar dependence on BMI was observed for BARD and FIBC3 [Supplementary Figure 2 (36)], while BMI did not affect FIB-4 (Fig. 1E and 1F), HFS, APRI, or ADAPT [Supplementary Figure 2 (36)]. We also examined NFS in patients with T2DM compared to those without [Supplementary Table 8 (36)]. The presence of T2DM associated with a lower overall specificity of NFS (61% vs 89%), but patients with T2DM were also significantly more obese (data not shown). Regarding fibrotic NASH, the diagnostic performance of MACK-3 was unaffected by BMI [Supplementary Figure 3 (36)].

### BMI-adjusted Cutoffs Improve the Diagnostic Performance of NFS for Advanced Fibrosis

**Figure 2.**
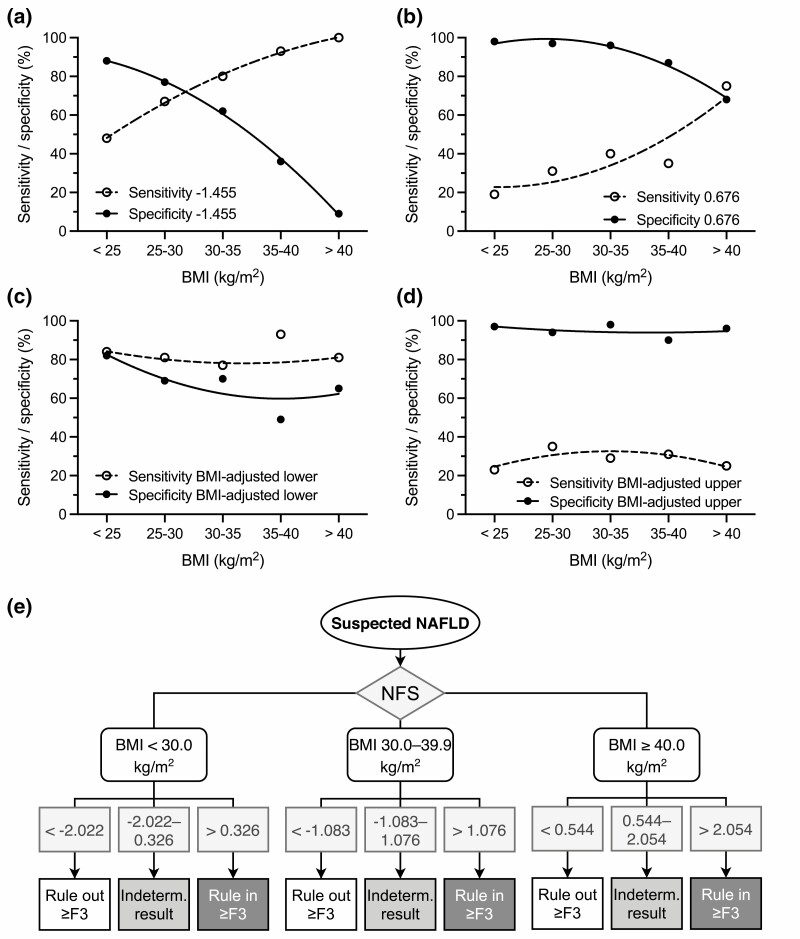
Body mass index (BMI)-adjusted cutoffs improve the performance of the NAFLD Fibrosis Score (NFS) for advanced fibrosis in all patients (n = 1232). Sensitivities and specificities for the (A) lower and (B) upper cutoffs of NFS using the standard cutoffs of −1.455 and 0.676 for advanced (F3-F4) fibrosis and for the BMI-adjusted (C) lower and (D) upper cutoffs in groups divided based on the degree of obesity. Black circles and solid lines denote specificity, and white circles and dashed lines denote sensitivity. Regression lines were fitted using a quadratic model for visualization purposes. (E) Schematic illustration of the use of BMI-adjusted NFS cutoffs to either rule in or rule out advanced fibrosis. Patients who have NFS between the upper and lower cutoffs are classified as indeterminate.

We next wished to characterize in detail the relationship between BMI and NFS. To this end, all patients (n = 1237) were divided into groups based on the degree of obesity (nonobese, BMI < 25.0 kg/m^2^, n = 160; overweight, BMI 25.0-29.9 kg/m^2^, n = 442; obese, BMI 30.0-34.9 kg/m^2^, n = 265; severely obese, BMI 35.0-39.9 kg/m^2^, n = 159; morbidly obese, BMI ≥ 40.0 kg/m^2^, n = 211). The AUROCs of NFS to diagnose advanced fibrosis were similar across the BMI groups [Supplementary Figure 4, Supplementary Table 9 (36)]. The lower cutoff for NFS (−1.455) had a specificity of 88% in the group with BMI < 25 kg/m^2^, decreasing to 9% in the group with BMI ≥ 40 kg/m^2^ (Fig. 2A). Specificity of the upper cutoff (0.676) decreased from 98% to 68% (Fig. 2B). Correspondingly, sensitivities increased with BMI (Fig. 2A and 2B). The other composite scores performed similarly as in the overweight/obese cohort [Supplementary Figures 4 and 5 (36)].

**Figure 3.**
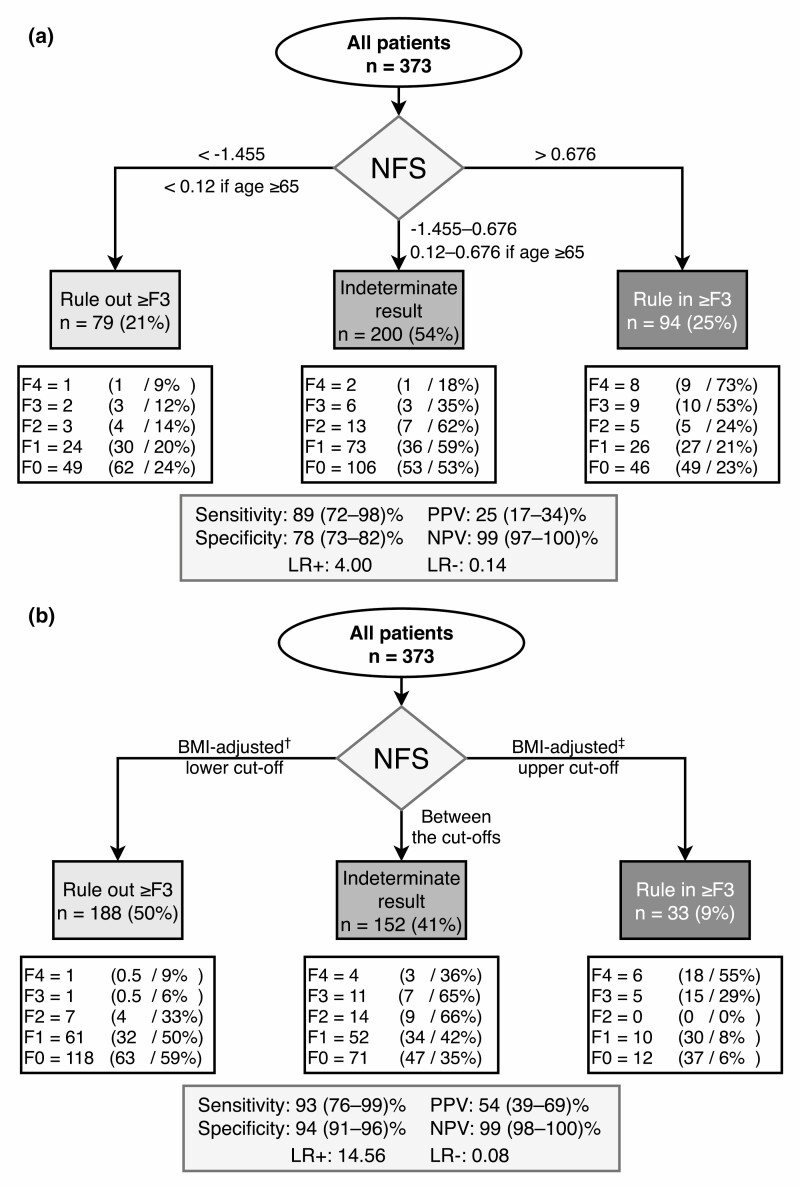
Use of body mass index (BMI)-adjusted cutoffs for the NAFLD Fibrosis Score (NFS) significantly improves diagnostic performance in the overweight/obese cohort (n = 373). Flowcharts illustrating use of either the (A) standard or (B) BMI-adjusted cutoffs of NFS to identify advanced fibrosis (F3-F4). In white rectangles are shown the allocation of patients with different stages of fibrosis (F0-F4) into low (rule out ≥F3), high (rule in ≥F3), and indeterminate risk categories. Percentage values separated by slashes indicate the proportion of patients in the risk category as well as the proportion out of all patients having the same fibrosis stage. ^†^−2.022 (BMI < 30.0 kg/m^2^), −1.083 (BMI 30.0-39.9 kg/m^2^), 0.544 (BMI ≥ 40 kg/m^2^). ^‡^0.326 (BMI < 30.0 kg/m^2^), 1.076 (BMI 30.0-39.9 kg/m^2^), 2.054 (BMI ≥ 40 kg/m^2^). Abbreviations: LR+, positive likelihood ratio; LR-, negative likelihood ratio; NPV, negative predictive value; PPV, positive predictive value.

Because the established cutoffs for NFS performed especially poorly in obese and morbidly obese individuals, we determined optimal cutoffs to either rule in or rule out advanced fibrosis separately in nonobese (BMI < 30.0 kg/m^2^), obese (BMI 30.0-39.9 kg/m^2^), and morbidly obese (≥40.0 kg/m^2^) patients (Table 4). The groups were similar with respect to clinical variables (data not shown) and the AUROCs of NFS (Table 4). When finally applied in all patients, the new BMI-adjusted cutoffs markedly improved the sensitivity and specificity of NFS over the entire range of BMI (Fig. 2C and 2D, Table 5). Those left in the indeterminate range comprised 31% to 39%. The negative likelihood ratio to rule out advanced fibrosis was 0.25 to 0.29, and the positive likelihood ratio to rule in, 5.17 to 7.50, confirming the applicability of these cutoffs in clinical practice. Figure 2E illustrates use of the BMI-adjusted cutoffs. In the overweight/obese cohort, compared to the old cutoffs (Fig. 3A), use of the new cutoffs resulted in significantly higher specificity (78% vs 94%, *P* < 0.001) and PPV (25% vs 54%, *P* < 0.001), as well as in fewer patients classified as indeterminate (54% vs 41%) [Fig 3B, Supplementary Table 10 (36)].

**Table 4.** Derivation of new BMI-adjusted cutoffs for NFS to identify advanced fibrosis in all patients with available NFS (n = 1232)

Group	n	F3-F4, n	AUROC (95% CI)	Cutoff	Se, %	Sp, %	PPV, %	NPV, %	Interpretation
BMI < 30.0 kg/m^2^									
Derivation	421	81	0.81 (0.76-0.87)	−2.022	85	58	32	94	Rule out
				0.326	30	95	57	85	Rule in
				−1.309	78	78	45	94	Best single
Validation	179	34	0.85 (0.78-0.92)	−2.022	85	62	35	95	Rule out
				0.326	38	96	68	87	Rule in
				−1.309	79	77	45	94	Best single
BMI 30.0-30.9 kg/m^2^									
Derivation	296	66	0.79 (0.73-0.85)	−1.083	85	58	37	93	Rule out
				1.076	33	95	65	83	Rule in
				−0.438	73	73	43	90	Best single
Validation	127	28	0.81 (0.71-0.91)	−1.083	86	61	38	94	Rule out
				1.076	21	98	75	82	Rule in
				−0.438	71	77	47	91	Best single
BMI ≥ 40.0 kg/m^2^									
Derivation	146	11	0.77 (0.64-0.89)	0.544	82	67	17	98	Rule out
				2.054	28	95	30	94	Rule in
				0.544	82	67	17	98	Best single
Validation	63	5	0.83 (0.65-1.00)	0.544	80	60	15	97	Rule out
				2.054	40	91	29	95	Rule in
				0.544	80	60	15	97	Best single

Abbreviations: AUROC, area under the receiver operating characteristic; BMI, body mass index; NFS, NAFLD Fibrosis Score; NPV, negative predictive value; PPV, positive predictive value; Se, sensitivity; Sp, specificity.

**Table 5.** Application of the new BMI-adjusted cutoffs for NFS to rule in or rule out advanced fibrosis in all patients with available NFS (n = 1232)

NFS value	n	F0-F2, n	F3-F4, n	Se, %	Sp, %	PPV, %	NPV, %	LR+	LR−	Interpretation
BMI < 30.0 kg/m^2^										
<−2.022	303	286	17	85	59	33	94	2.07	0.25	Rule out
−2.022-0.326	236	175	61	–	–	–	–	–	–	Indeterminate
>0.326	61	24	37	32	95	61	86	6.40	0.72	Rule in
≥−1.309	199	109	90	78	78	45	94	3.55	0.28	Best single
BMI 30.0-39.9 kg/m^2^										
<−1.083	208	194	14	85	59	37	93	2.07	0.25	Rule out
−1.083-1.076	173	121	52	–	–	–	–	–	–	Indeterminate
>1.076	42	14	28	30	96	67	83	7.50	0.73	Rule in
≥−0.438	154	86	68	72	74	44	90	2.77	0.38	Best single
BMI ≥ 40 kg/m^2^										
<0.544	128	125	3	81	65	16	98	2.31	0.29	Rule out
0.544-2.054	64	56	8	–	–	–	–	–	–	Indeterminate
>2.054	17	12	5	31	94	29	94	5.17	0.73	Rule in
≥0.544	81	68	13	81	65	16	98	2.31	0.29	Best single

Abbreviations: BMI, body mass index; LR+, positive likelihood ratio; LR−, negative likelihood ratio; NFS, NAFLD Fibrosis Score; NPV, negative predictive value; PPV, positive predictive value; Se, sensitivity; Sp, specificity.

### Sequential Testing Increases Diagnostic Yield for Advanced Fibrosis

**Figure 4.**
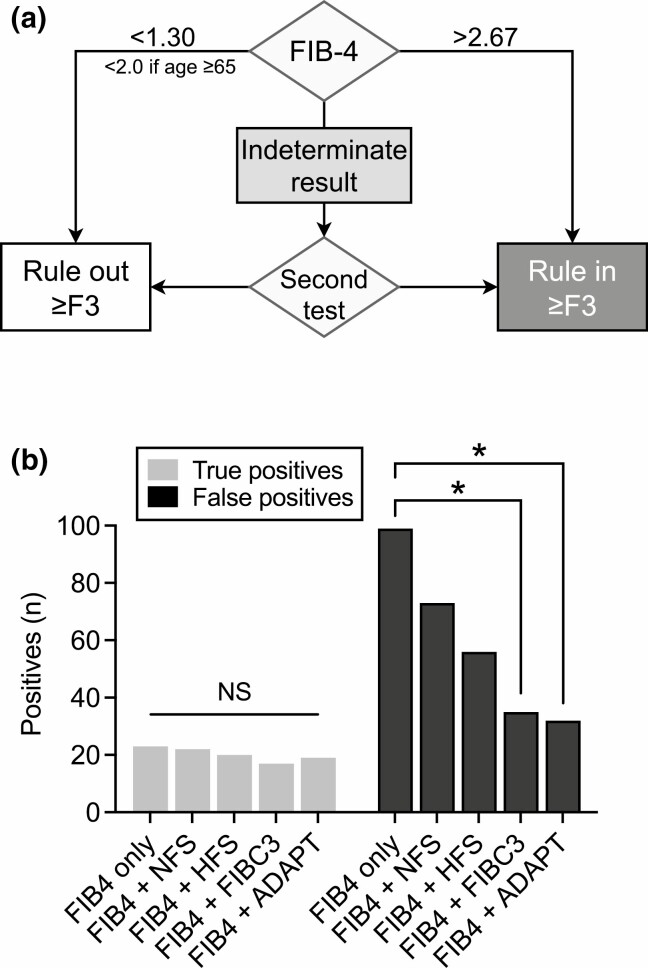
Sequential use of the Fibrosis-4 Index (FIB-4) followed by another biomarker increases the diagnostic yield for advanced fibrosis. (A) Proposed algorithm to test patients with FIB-4 in the indeterminate range. (B) Accuracy of using FIB-4 alone compared to sequential use with either the NAFLD Fibrosis Score (NFS), the Hepamet Fibrosis Score (HFS), FIBC3, or ADAPT to identify advanced fibrosis in the overweight/obese cohort (n = 378). Cutoffs used for NFS were BMI-adjusted as follows: −2.022 (BMI < 30.0 kg/m^2^), −1.083 (BMI 30.0-39.9 kg/m^2^), and 0.544 (BMI ≥ 40 kg/m^2^); cutoffs used for the other scores: HFS, 0.12; FIBC3, 0.4; and ADAPT, 6.3287. Light gray bars show true positives and dark gray bars false positives. The Chi-squared test was used. *P < 0.05. Abbreviation: NS, not significant.

Finally, we tested in the overweight/obese cohort whether sequential use of 2 composite scores improves accuracy to diagnose advanced fibrosis over a single score. We chose FIB-4 as the initial test (Fig. 4A) as it had an adequate discriminatory ability by itself, was independent of BMI, and is already in widespread use. An algorithm beginning with FIB-4 and followed by ADAPT for those with FIB-4 in the indeterminate range showed highest diagnostic performance (Table 6). It classified 51/378 (13%) patients as high risk and had the highest PPV (37%) and specificity (91%), which were significantly higher than those of FIB-4 alone (*P* < 0.0001). The specificity was also higher than that of ADAPT alone (*P* < 0.01), while sensitivity was similar (*P* = 0.25). An algorithm with FIB-4 followed by FIBC3 performed comparably (Table 6). When used in sequence after FIB-4, ADAPT and FIBC3 significantly reduced the degree of false-positive diagnoses while maintaining similar sensitivity to FIB-4 alone (Fig. 4B). Supplementary Figures 6 to 9 (36) illustrate the studied algorithms.

**Table 6.** Diagnostic performance of sequential algorithms to identify advanced fibrosis (F3-F4) in the overweight/obese cohort (n = 378)

Algorithm	Second test cutoff	Se, % (95% CI)	Sp, % (95% CI)	PPV, % (95% CI)	NPV, % (95% CI)	LR+	LR−	NND
FIB-4 + ADAPT	6.3287	68 (48-84)	91 (87-94)	37 (24-52)	97 (95-99)	7.56	0.35	1.7
FIB-4 + FIBC3	0.4	61 (41-78)	90 (86-93)	33 (20-47)	97 (94-98)	6.10	0.43	2.0
FIB-4 + HFS^a^	0.12	71 (51-87)	84 (79-87)	26 (17-38)	97 (95-99)	4.44	0.35	1.8
FIB-4 + NFS^b^	BMI-adjusted^c^	82 (63-94)	78 (72-81)	23 (15-31)	98 (96-99)	3.73	0.23	1.7

Abbreviations: FIB-4, Fibrosis-4 Index; HFS, Hepamet Fibrosis Score; LR+, positive likelihood ratio; LR−, negative likelihood ratio; NFS, NAFLD Fibrosis Score; NND, number needed to diagnose; NPV, negative predictive value; PPV, positive predictive value; Se, sensitivity; Sp, specificity.

^a^n = 371.

^b^n = 373.

^c^BMI < 30.0 kg/m^2^: −2.022; BMI 30.0-39.9 kg/m^2^: −1.083; ≥40.0 kg/m^2^: 0.544.

## Discussion

We examined both direct fibrosis biomarkers and composite scores in an obese cohort, which is hitherto the most comprehensive in terms of its size and the number of biomarkers studied. The best composite scores for advanced fibrosis were ADAPT and FIB-4, while the most accurate tests for fibrotic NASH were MACK-3 and ADAPT. We observed that obesity highly influenced the performance of NFS, BARD, and FIBC3, all of which include BMI in their formulae. The specificity of NFS, which is recommended as a first-line test in several guidelines (8,43,44), substantially deteriorated as a function of BMI. This finding is important given the high proportion of patients with NAFLD who are obese. We established new BMI-adjusted cutoffs for NFS, restoring its utility over a wide range of adiposity.

The validity of blood-based fibrosis biomarkers in morbidly obese cohorts has, to the best of our knowledge, previously been addressed by 6 studies (17-22). Only one of these compared multiple blood-based tests, and none included data on neo-epitope biomarkers. Our overweight/obese cohort from a single center was large, as it included 378 individuals with 11 biomarkers. The highest combined sensitivity and specificity for advanced fibrosis was possessed by the PRO-C3-dependent score ADAPT. It also had a similar AUROC as MACK-3 to identify fibrotic NASH, although it lacks validated cutoffs. Interestingly, PRO-C3 was inferior to FIB-4 in identifying advanced fibrosis, consistent with recent reports on its limited utility when used alone (14). The high accuracy of ADAPT, which incorporates PRO-C3 as a direct measurement of extracellular matrix formation, emphasizes the importance and utility of assessing markers of extracellular matrix turnover in addition to other liver-related tests. Regarding the other composite scores, the suggested cutoff of 1.0 for APRI resulted in high specificity but poor sensitivity, making it useful only as a rule-in test (45). BARD had the lowest AUROC and had low specificity, mirroring previous findings in obese individuals (21). These considerations support use of ADAPT or the inexpensive FIB-4, which has limitations regarding its use of 2 cutoffs, as first-line tests in obese individuals. Even better, as discussed later, sequentially combining 2 tests might be the ideal and most cost-effective solution.

The AUROCs of every biomarker were similar at all degrees of adiposity. This is in keeping with baseline data from the STELLAR trial, in which BMI did not affect the AUROCs of FIB-4 or NFS (46). The limitation of only considering AUROC, however, is that it is a measure merely reflecting the overall discriminatory capacity of a diagnostic test (47). Real-world test performance is determined by applying the selected cutoff values and judged by sensitivity, specificity, predictive values, and likelihood ratios. Previous studies have failed to examine the relationship between these metrics and BMI (17-22,46). As NFS, BARD, and FIBC3 all incorporate BMI as a predictor variable, we hypothesized that they would suffer in specificity in obese individuals. This was true for all 3 biomarkers. Of the BMI-dependent biomarkers, FIBC3 was least affected, probably due to incorporating PRO-C3 and the smaller weighting of BMI in its formula.

Our main finding was the strong dependence, especially of the widely used NFS score, on BMI. For the standard cutoffs of NFS, specificity began sharply declining in those with BMI over 30 kg/m^2^, with a concomitant increase in sensitivity. Interestingly, sensitivity and specificity of the lower cutoff value (−1.455) in lean individuals were similar to those of the higher cutoff value (0.676) in the morbidly obese (Fig. 2A and 2B). This implies that the cutoffs should be adjusted upward to prevent a high rate of false-positive classifications in individuals with a high BMI. We found that separate cutoffs are required for lean-overweight (BMI < 30.0 kg/m^2^), obese (BMI 30.0-30.9 kg/m^2^), and morbidly obese patients (BMI ≥ 40.0 kg/m^2^). The new cutoffs restored the specificity and sensitivity of NFS in each BMI group, approximating those previously reported in leaner cohorts (48). We did not consider developing such new cutoffs for BARD due to its low AUROC in these patients.

Similar to many other composite scores, NFS was developed by using stepwise logistic regression (7), an algorithm that easily leads to overfitting and not necessarily selecting the most meaningful combination of variables to predict disease state (ie, advanced fibrosis) (49). We surmise that the weight given to BMI in the NFS formula may be too high. Furthermore, the relationship between fibrosis risk and BMI may not be linear, especially in the severely obese range. These notions are corroborated by the slight but consistent outperforming of NFS by the much simpler FIB-4 (45,50). Recent data also suggest that, at the general population level, the ability of NFS to predict incident severe liver disease is inferior to that of FIB-4 among the obese (51). In addition to age, FIB-4 only includes the aspartate-to-alanine aminotransferase ratio as well as platelets, which are among the best indicators of advanced fibrosis and cirrhosis in routine laboratory tests (52). Of interest, McPherson et al showed that higher cutoffs for FIB-4 and NFS are needed for individuals aged ≥65 years (37). The effect of age on the biomarkers is partly explainable by changes exerted by aging on transaminases and partly by the inclusion of age as a predictor variable (37). Thus, as epidemiological risk factors such as BMI do not truly increase liver fibrosis in a dose-dependent manner, including them in predictive scores may lead to poor specificity in populations with a high prevalence of those risk factors.

Despite the paucity of data regarding the cost-effectiveness of population-scale screening efforts (25), the European guidelines recommend that at-risk individuals be screened for advanced fibrosis (8). To prevent an overburdening of liver clinics as well as unnecessary liver biopsies, sequential use of blood-based tests has been proposed (25,27). We found that sequentially combining FIB-4 with either ADAPT or FIBC3 significantly improved specificity to diagnose advanced fibrosis in the overweight/obese cohort (Fig. 4, Table 6). FIB-4 is an ideal initial test as it is easily calculated, inexpensive, and has consistently shown high accuracy for advanced fibrosis (9,50,53). Srivastava et al prospectively evaluated a primary care pathway for NAFLD in which patients with an indeterminate result by FIB-4 were further evaluated using the enhanced liver fibrosis test. This test incorporates concentrations of hyaluronic acid, tissue inhibitor of matrix metalloproteinases-1, and amino-terminal propeptide of type III procollagen, which are direct biomarkers akin to CK-18 and PRO-C3, respectively. During a 2-year period, the studied pathway reduced unnecessary referrals by 80% while improving the detection of advanced fibrosis approximately 5-fold (28). Notably, the inclusion of only patients with elevated liver enzymes in this study likely contributed to the high diagnostic yield. A separate investigation deemed the pathway more cost-effective as compared to screening of fibrosis using elastography (54). Similar to findings by Srivastava et al, use of ADAPT after FIB-4 in the present study reduced potential unnecessary referrals (false positives) by as much as 68% (Fig. 4).

A limitation of our study was the relatively small albeit epidemiologically representative prevalence of advanced fibrosis in the overweight/obese cohort. This translated into wide CIs for test sensitivity. Moreover, in validating BMI-adjusted cutoffs, we were unable to devise separate cutoffs for individuals aged 65 years or older due to a low number of patients in this age range. Thus, we cannot recommend using the new cutoffs in this age category. As the study population included patients solely of the European ancestry, our findings may be inapplicable to other ethnicities due to different relationships between liver disease, obesity, and metabolic risk factors (55). We also acknowledge that visceral adiposity may be a better indicator of fibrosis risk compared to BMI (56). In the overweight/obese cohort, however, body composition data were unavailable. Lastly, our study was cross-sectional, making it difficult to reconcile how the proposed sequential algorithms or BMI-adjusted cutoffs potentially affect diagnostic yield in real-life referral pathways. Future studies should validate the new NFS cutoffs in completely independent cohorts, in patients aged ≥65 years, and preferably with longitudinal outcome data to ascertain performance in this application.

In summary, we found the best-performing fibrosis scores in obese individuals to be FIB-4 and ADAPT, both of which were unaffected by adiposity. The inexpensive FIB-4 is currently more feasible as it is simple, easily calculated in the clinical practice, and performs slightly better than comparable biomarkers in head-to-head comparisons (50). FIB-4 can therefore be recommended to be used by all clinicians for screening of advanced liver fibrosis in patients with obesity. In cases where NFS has been adopted as the primary test, however, we recommend using BMI-adjusted cutoffs. Sequentially combining FIB-4 with the PRO-C3-incorporating ADAPT or FIBC3 scores may significantly reduce the degree of false-positive diagnoses, potentially reducing screening-related costs and healthcare burden.

## Data Availability

Restrictions apply to the availability of some or all data generated or analyzed during this study to preserve patient confidentiality or because they were used under license. The corresponding author will on request detail the restrictions and any conditions under which access to some data may be provided.
